# Afterload pressure and left ventricular contractility synergistically affect left atrial pressure during veno-arterial ECMO

**DOI:** 10.1016/j.jhlto.2023.100044

**Published:** 2023-12-14

**Authors:** Jacky Jiang, Pankaj Jain, Audrey Adji, Michael Stevens, Gabriel Matus Vazquez, Sumita Barua, Sambavan Jeyakumar, Christopher Hayward

**Affiliations:** a University of New South Wales, Sydney, Australia; b St Vincent’s Hospital Centre for Applied Medical Research, Sydney, Australia; c Victor Chang Cardiac Research Institute, Sydney, Australia

**Keywords:** veno-arterial extra-corporeal membrane oxygenation (VA-ECMO), mock circulatory loop (MCL), cardiogenic shock, left ventricular distension, pulmonary edema

## Abstract

**Background:**

Veno-arterial extra-corporeal membrane oxygenation (VA-ECMO) may cause adverse effects including increased left ventricular (LV) filling pressure, LV distension, and pulmonary edema. We aimed to quantify the effects of ECMO flow, LV contractility, aortic pressure (AoP), and ECMO configuration on left atrial pressure (LAP) during VA-ECMO for cardiogenic shock in a mock circulatory loop (MCL).

**Methods:**

An MCL simulated a normal state, LV failure, right ventricular failure, and biventricular failure. The ECMO return cannula was placed in the femoral artery (retrograde flow) or ascending aorta (antegrade flow). ECMO flow was incrementally increased from 0 to 5 liter/min. LAP, mean AoP, ECMO flow, and total cardiac output were measured at steady state.

**Results:**

During VA-ECMO, LAP increased linearly with AoP, with the slope greater in the presence of LV impairment compared to preserved LV function. When AoP was held constant, as is the goal of therapy in clinical management, ECMO flow had no effect on LAP. In multivariable linear regression, AoP and LV contractility (*p* < 0.001 for each) correlated independently with LAP, but ECMO flow did not. ECMO return flow direction had no effect on LAP.

**Conclusions:**

AoP and LV contractility, but not circuit flow or direction, independently determine LAP under VA-ECMO support. By controlling each of these inputs, vasodilator and inotrope management may combine synergistically to prevent VA-ECMO-related complications.

## Graphical abstract

**Figure:**
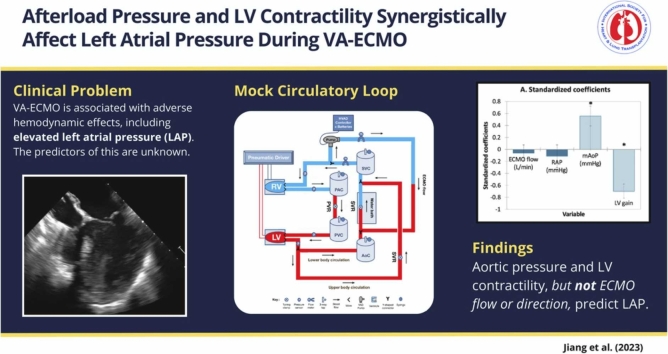

## Background

Veno-arterial extra-corporeal membrane oxygenation (VA-ECMO) is a cornerstone in the management of patients with severe cardiogenic shock.^1^, ^2^, ^3^ Despite its use having increased substantially, survival with VA-ECMO remains poor with approximately 40% of patients surviving to hospital discharge.^1^

While VA-ECMO is able to provide full cardiopulmonary support, it is also associated with a range of adverse hemodynamic and clinical effects, including left ventricular (LV) distension, thrombus formation, and pulmonary edema.^4^ These effects are thought to be primarily driven by increased afterload due to retrograde flow from the VA-ECMO return cannula, resulting in reduced LV stroke volume and subsequent elevation of diastolic LV pressure and volume.^5^, ^6^, ^7^ Important questions remain unanswered however, including whether afterload and LV contractility have synergistic effects; whether the direction of ECMO return flow is significant; and whether ECMO flow itself is an independent determinant of LV filling pressure.

Several measures are already utilized in the clinical setting to mitigate the risk of LV distension and its associated complications. These range from simple measures, such as reducing ECMO flow and administration of vasodilators and/or inotropes, to progressively more invasive and costly measures including intra-aortic balloon pump, transvalvular microaxial ventricular assist devices, and direct surgical “venting” of the LV.^7^, ^8^ A more complete understanding of the effects of hemodynamic and VA-ECMO operating conditions on LV preload is necessary to ensure optimal efficacy and resource utilization associated with these measures.

Mock circulatory loops (MCLs) allow for precise control of a number of hemodynamic and circuit-related variables and systematic assessment of the interactions between mechanical circulatory support devices and the cardiovascular systems, and have previously been used to investigate the effects of different VA-ECMO unloading techniques and cannula properties.^9^, ^10^, ^11^, ^12^, ^13^ We sought to utilize an MCL to systematically assess the effects of changes in afterload pressure, ventricular contractility, and ECMO flow and direction on left atrial pressure (LAP) during VA-ECMO support.

## Methods

### Mock circulatory loop design

The MCL used in this study has been previously described in detail by Shehab et al.^13^ Briefly, the left and right ventricles were constructed using a truncated ellipsoid-shaped deformable silicon diaphragm encased inside a transparent sealed air chamber. Ventricular contractility was achieved by external electropneumatic (EP) compression using a proprietary programmed air delivery system (Simulink, The MathWorks Inc, Natick MA). The EP regulators were programmed to deliver cyclic air pressure waveforms to each ventricle to create realistic systolic and diastolic pressures. These waveforms were then scaled by a multiplier (LV and RV Gain) to achieve pathologic ventricular function. Four identical hermetically sealed, cylinder-shaped Windkessel chambers (100 mm diameter × 320 mm height) were used to simulate aortic, systemic venous, pulmonary arterial, and pulmonary venous compliance. The circuit in this study included separate upper and lower body systemic circulations. Systemic vascular resistance (SVR) and pulmonary vascular resistance (PVR) were created using tuning clamps placed on the systemic and pulmonary arterial limbs, respectively. The MCL was filled with 5 liter of 40%wt to 60%wt glycerol-water solution, maintained at 37°C by a JB Nova JBN12 Water Bath (Grant Instruments Ltd, Cambridge, UK) to simulate the viscosity of blood with a hematocrit of 34%.

**Figure 1:**
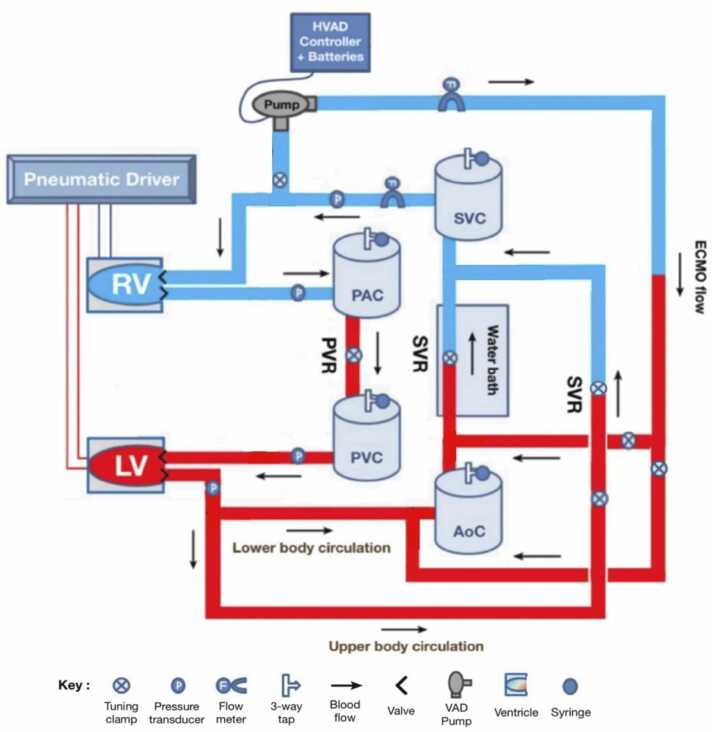
Mock circulatory loop schematic. AoC, aortic compliance; ECMO, extracorporeal membrane oxygenation; HVAD, HeartWare ventricular assist device; LV, left ventricle; PAC, pulmonary arterial compliance; PVC, pulmonary venous compliance; PVR, pulmonary vascular resistance; RV, right ventricle; SVC, systemic venous compliance; SVR, systemic vascular resistance; VAD, ventricular assist device.

The MCL included an ECMO circuit consisting of Tygon tubing, draining from the right atrium and with arterial return limbs to the femoral artery and proximal aorta. Tuning clamps were placed on each arterial return limb, allowing for switching between retrograde and antegrade ECMO flow. A HeartWare ventricular assist device (HVAD) (Medtronic, Minneapolis, MN) was placed within the circuit to simulate the centrifugal pump in an ECMO system. This HVAD was connected to a controller and monitor which allowed for adjustment of pump speed from 1,800 to 4,000 revolutions per minute. A schematic of the MCL in conventional VA-ECMO configuration is shown in Figure 1.

ECMO flow and cardiac output were measured by BioProTT Clamp-On Transducers (em-tec GmbH, Finning, Germany) one placed distal to the HVAD pump within the ECMO circuit, and the other between the systemic venous compliance chamber and the right ventricle. Aortic pressure (AoP), LAP, and right atrial pressure (RAP) were measured using fluid-filled pressure transducers.

### Experimental method

The MCL was run in steady state, with real-time flow waveforms and pressures monitored by dSpace ControlDesk (dSpace GmbH, Paderborn, Germany). Four cardiac conditions were simulated: (1) normal function (LAP and RAP between 5 and 10 mm Hg), (2) left ventricular failure (LAP > 20 mm Hg, RAP < 10 mm Hg), (3) right ventricular failure (RVF) (LAP < 10 mm Hg, RAP > 20 mm Hg), and (4) biventricular failure (LAP and RAP > 20 mm Hg). These conditions were achieved by altering LV and RV contractilities, SVR, PVR, and compliances. Heart rate was maintained at 60 beats per minute. Mean AoP prior to commencement of VA-ECMO flow was set to either 50, 70, or 90 mm Hg by adjusting SVR. In each experiment, ECMO flow was varied by adjusting HVAD pump speed, to targets of 0, 2, 3, 4, and 5 liter/min.

Four experiments were performed. In experiment 1, ECMO flow was incrementally increased while SVR, while compliances and contractility were kept constant within each heart failure state. In experiment 2, SVR was adjusted to maintain constant AoP with increasing ECMO flow. In experiment 3, LV contractility was incrementally reduced from normal to severely impaired by scaling down the voltage delivered to the LV EP by 33%. In experiment 4, return flow was adjusted from retrograde to antegrade while resistances, compliances, and contractility were kept constant. Table S1 in the Supplementary Appendix summarizes these experiments, and the specific combinations of heart failure state, ECMO configuration, ECMO flow direction, mean AoP, and ECMO flow rate examined in each.

### Data acquisition and analysis

Flow and pressure waveforms were recorded using dSpace ControlDesk while pump speed was recorded from the HVAD controller. All data were processed in MATLAB (MathWorks, Natick, MA) and analyzed using SPSS Statistics 29 (IBM, Armonk, NY). The means of the pressures and flows within each experiment were calculated and used for comparison. The primary dependent variable of interest was LAP.

To compare means between groups, paired *t*-test or 1-way analysis of variance were used as appropriate. Simple linear regressions were used to ascertain relationships between continuous variables. Multiple linear regression was used to determine independent predictors of the dependent variable. For all statistical tests, a *p*-value less than 0.05 was considered statistically significant.

## Results

### Left atrial pressure and aortic pressure

**Figure 2:**
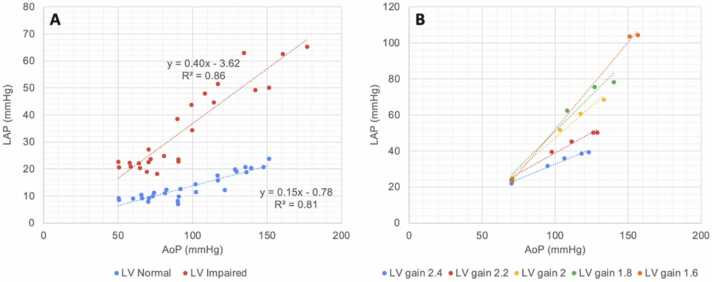
Relationship between left atrial pressure and aortic pressure. (A) Experiment 1 data stratified by left ventricular function (normal vs impaired). (B) Experiment 3 data stratified by left ventricular contractility. Higher gain corresponds to greater contractility. AoP, aortic pressure; LAP, left atrial pressure; LV, left ventricle.

The relationship between LAP and AoP is illustrated in Figure 2. In Figure 2A, data from experiment 1 are divided in binary fashion, into “LV Normal” (comprising “Normal” and “RVF” states) and “LV Impaired” (comprising left ventricular failure and biventricular failure states). Within each subgroup, LAP increases linearly with increased AoP (*r*^2^ =0.814 for LV Normal; *r*^2^ =0.855 for LV impaired, *p* < 0.001 for each). The slope of this relationship is nearly 3-fold higher for the LV Impaired subgroup (0.41) compared to the LV Normal subgroup (0.15). In experiment 3, LV contractility was adjusted incrementally from normal (LV Gain 2.4) to severely impaired (LV Gain 1.6). There is a corresponding increase in the slope of the LAP-AoP relationship (Figure 2B), consistent with effect modification of contractility on this linear relationship.

### Left atrial pressure and ECMO flow rate

**Figure 3:**
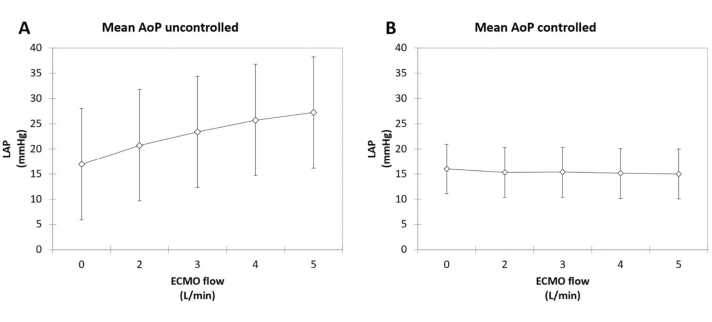
Relationship between VA-ECMO flow and left atrial pressure. (A) Experiment 1 data showing numerical increase in LAP with increased ECMO flow when aortic pressure is not controlled. (B) Experiment 2 data showing no increase in LAP with increased ECMO flow when aortic pressure is held constant. Error bars represent 95% confidence intervals. AoP, aortic pressure; ECMO, extracorporeal membrane oxygenation; LAP, left atrial pressure.

The relationship between LAP and VA-ECMO flow rate is summarized in Figure 3. When SVR is held constant and mean AoP is uncontrolled, increased ECMO flow is associated with a numerical but not statistically significant increase in LAP (*p* = 0.684, Figure 3A). However, when SVR is varied to allow constant mean AoP with increased ECMO flow, there is no corresponding increase in LAP (*p* = 0.999, Figure 3B).

### Left atrial pressure and right atrial pressure

**Figure 4:**
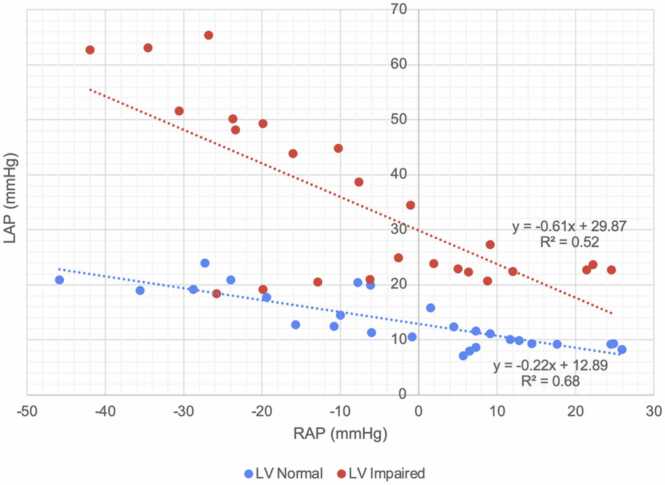
Relationship between left atrial pressure and right atrial pressure. Experiment 1 data stratified by left ventricular function (normal vs impaired). LAP, left atrial pressure; LV, left ventricle; RAP, right atrial pressure.

In experiment 1, LAP decreased linearly as RAP increased (*r*^2^ = 0.327, *p* < 0.001). When the data were stratified according to LV function, both “LV Normal” and “LV impaired” subgroups demonstrated a negative, linear association between LAP and RAP (*r*^2^ = 0.682 for LV normal; *r*^2^ = 0.523 for LV impaired, *p* < 0.001 for both). The slope of this linear relationship was steeper in the LV impaired subgroup (−0.60 vs −0.22) consistent with greater sensitivity of the impaired LV to changes in RAP. This is summarized in Figure 4.

### Retrograde and antegrade VA-ECMO flow

The effects of ECMO return flow direction on LAP were explored in experiment 4. There was no significant difference between retrograde and antegrade return flow in terms of LAP (*p* = 0.11), mean AoP (*p* = 0.06), or total cardiac output (*p* = 0.06). There was a statistically but not clinically significant increase in ECMO flow with antegrade return compared to retrograde return (3.5 vs 3.4 liter/min, *p* = 0.001).

### Multivariable model to predict LAP

**Figure 5:**
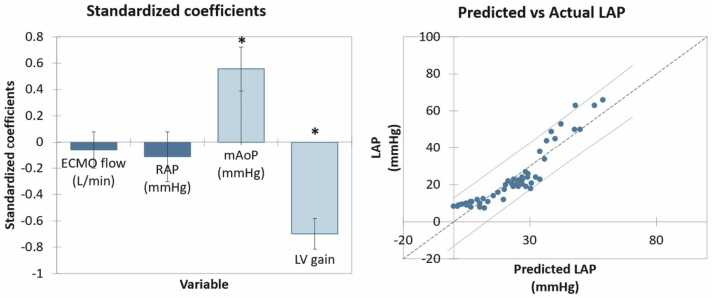
Multivariable linear regression model to predict LAP. (A) Standardized coefficients. AoP and LV gain were independent predictors of LAP. (B) Predicted vs actual LAP using the regression model. Error bars and lines represent 95% confidence intervals. ECMO, extracorporeal membrane oxygenation; LAP, left atrial pressure; LV, left ventricular; mAoP, mean aortic pressure; RAP, right atrial pressure. *p < 0.001.

A multiple linear regression model using data from experiment 1 incorporated AoP, LV gain, ECMO flow rate, and RAP as independent variables. AoP (β = 0.556, *p* < 0.001) and LV gain (β = −0.695, *p* < 0.001), but not RAP (β = −0.114, *p* = 0.242) or ECMO flow rate (β = −0.069, *p* = 0.326) were independent predictors of LAP (*r*^2^ for model = 0.852, *p* < 0.001). These results are summarized in Figure 5.

## Discussion

Despite its role as a cornerstone of the management of severe cardiogenic shock, VA-ECMO continues to be associated with a high mortality and complication rate. Complications associated with impaired LV ejection and elevated LV filling pressure—including thrombus formation and pulmonary edema—are well recognized. Understanding the hemodynamic and circuit-related factors that contribute to elevation of LV filling pressure is critical to the rational, pre-emptive application of mitigation strategies, including mechanical unloading devices. In the absence of hemodynamic data beyond limited animal studies and in-silico simulations, use of these devices is guided by expert opinion. This has resulted in a high degree of interinstitutional variability and a sharp recent increase in the use of percutaneous left ventricular assist devices with their associated increased cost and complication rates.^7^ More robust physical data—in-vitro and in-vivo—are needed.

In this context, the primary findings of this MCL study are (1) under VA-ECMO support, AoP, and LV contractility independently correlate with LAP; (2) the effect of AoP is mediated by LV contractility; (3) VA-ECMO flow rate does not independently affect LAP; and (4) direction of VA-ECMO flow does not affect LAP.

### AoP and LV contractility independently predict LAP during VA-ECMO

Using an in-silico model, Dickstein^6^ demonstrated that, assuming a constant Frank-Starling relationship, the increase in pulmonary capillary wedge pressure following VA-ECMO initiation is dependent on both baseline LV function and the degree of AoP elevation. In our study, LV contractility and AoP were independent predictors of LAP, confirming the findings of Dickstein in a physical model of the circulation. Of note, the consistency of findings between Dickstein’s model and ours occurred despite our model not incorporating Frank-Starling forces.

Our results extend those of Dickstein,^6^ by demonstrating an incremental steepening of the slope of the linear AoP-LAP relationship with reduction in LV contractility. These findings suggest that LV contractility acts as an effect modifier on the AoP-LAP relationship, pointing to a synergistic effect between LV contractility and AoP on the risk of elevated LV filling pressure. In the clinical setting, these results highlight the important role of inotropes and vasodilators—in combination where possible—to reduce LV pressures on VA-ECMO support. In our study, the slope of the AoP-LAP relationship in the setting of LV impairment was 0.40, suggesting that a reduction in AoP by 12.5 mm Hg is sufficient to achieve a clinically meaningful, 5 mm Hg reduction in LAP. Importantly, however, clinical use of vasodilators and inotropes as first-line therapies can be limited by vasoplegia or ongoing myocardial ischemia, respectively.^14^ It has been suggested that inotropic therapy may increase myocardial oxygen consumption in the setting of VA-ECMO, although this has yet to be demonstrated experimentally.^5^ In-vivo preclinical and clinical data are needed to determine a dose-response relationship between afterload and LV filling pressure reduction, and to determine the optimal target AoP and inotrope dose.

We found a statistically significant, negative linear relationship between LAP and RAP on univariate analysis, which was more pronounced in the setting of LV impairment. Given the lack of direct biventricular interaction in our MCL, this relationship is most likely due to the independent effects of ECMO flow directly on RAP, and indirectly on LAP via its effect on AoP. This is supported by the results of our multiple linear regression model, which showed no significant, independent relationship between LAP and RAP, but nevertheless requires confirmation in a dedicated MCL incorporating biventricular interactions.

### Flow rate and direction do not predict LAP during VA-ECMO

Ostadal et al^15^ showed, using a porcine model, that increasing ECMO flow in acute cardiogenic shock led to increases in systolic blood pressure, heart rate, and end-systolic volume, with decreases in LV stroke volume and ejection fraction resulting in a reduced cardiac output. This was the first study to report the flow-dependent effects of VA-ECMO on LV performance parameters. Similar conclusions were subsequently reached by Pavel et al,^16^ but in a porcine model of chronic heart failure. They found statistically significant decreases in LV peak pressure, end-diastolic pressure, and volume, end-systolic volume, and stroke work with increasing ECMO flow. However, both studies were unable to examine these effects while controlling for afterload pressure.

While our results did also show a numerical increase in LAP with increasing ECMO flow, when AoP was held constant, increasing ECMO flow no longer had any effect on LAP. Furthermore, in multivariable analysis, ECMO flow did not independently predict LAP. The putative effect of ECMO flow on LAP—demonstrated numerically in our study only when AoP was uncontrolled—can therefore be attributed solely to its effect on AoP. Changing the direction of ECMO return flow similarly had no effect on LAP. Taken together, these findings challenge the conventional orthodoxy that it is the retrograde nature of ECMO flow—and by extension, the amount of retrograde ECMO flow—that directly imparts afterload on the LV, therefore causing LV distension and increased filling pressure. Rather, consistent with the arguments of Dickstein,^6^ our study suggests it is increased AoP, *irrespective of the origin of this increase*, that primarily drives changes in filling pressure. Clinically, our findings suggest that, rather than reducing ECMO flow to reduce LAP as is standard practice, the same effect could be achieved simply through pharmacologic reduction in SVR without sacrificing ECMO flow.

### Limitations

This MCL was not designed to simulate complex regulatory adaptations, such as the baroreceptor reflex and Frank-Starling mechanism, nor the ability of VA-ECMO to produce adequate myocardial and peripheral tissue oxygenation and correct metabolic disturbance. Numerical models, hybrid MCLs, and in-vivo studies may more accurately represent these biological effects.^17^ Additionally, the ventricles in our MCL were connected “in-series”, without a common interventricular septum. Therefore, we were unable to examine the effects of ventricular interdependence, which may significantly attenuate the effects of VA-ECMO on LAP through changes in ventricular compliance.^17^, ^18^, ^19^, ^20^ Additionally, given these limitations of our MCL, the effects of changes in PVR and pulmonary capacitance on RAP and the LAP-RAP relationship in the setting of normal and impaired RV function were outside the scope of this study. This important component of the pump-patient interaction requires dedicated study in an MCL that incorporates direct biventricular interactions. Finally, we used a HeartWare HVAD in our VA-ECMO circuit instead of a dedicated ECMO pump. The HVAD is a reasonable surrogate for an ECMO pump, given that (1) it was placed in a right atrial-femoral artery configuration—identical to a peripheral VA-ECMO circuit; (2) HVAD is a centrifugal pump with similar engineering characteristics to a traditional ECMO pump; (3) we assessed hemodynamic responses only, and not oxygenation effects, and therefore did not require an oxygenator; and (4) we were able to achieve pump flows within an appropriate clinical range for a VA-ECMO circuit. Nevertheless, confirmation of our results using a dedicated VA-ECMO centrifugal pump is warranted.

### Implications and future directions

These findings highlight the critical role of afterload pressure and LV contractility in determining LV preload under VA-ECMO support. In terms of clinical translation, they highlight the critical and synergistic role of vasodilators and inotropes as first-line therapies to prevent pulmonary edema in this setting. Conversely, our results do not support the practice of altering ECMO flow to reduce LAP. Preclinical and clinical in-vivo studies are needed to establish a dose-response relationship of reduced afterload and positive inotropy on LAP, determine optimal therapeutic targets, and identify patients most likely to require escalation to more invasive measures, such as mechanical unloading.

## Conclusion

In this MCL study, LV function and afterload pressure, but not circuit flow or direction, synergistically affect LV filling pressure during VA-ECMO support. Further in-vivo studies are needed to confirm these findings and guide strategies to improve VA-ECMO outcomes.

## Ethics approval

This was an in-vitro mock circulatory loop study; no ethics approval was required.

## CRediT authorship contribution statement

All authors contributed to the study conception and design. Material preparation, data collection, and analysis were performed by Jacky Jiang, Pankaj Jain, Audrey Adji, Michael Stevens, Gabriel Matus Vazquez, Sumita Barua, and Christopher Hayward. The first draft of the manuscript was written by Jacky Jiang and Pankaj Jain, and all authors commented on previous versions of the manuscript. All authors read and approved the final manuscript.

## Disclosure statement

The authors declare the following financial interests/personal relationships which may be considered as potential competing interests: Christopher Hayward reports a relationship with Abbott Laboratories that includes consulting or advisory, nonfinancial support, speaking and lecture fees, and travel reimbursement. Christopher Hayward reports a relationship with Medtronic that includes consulting or advisory, nonfinancial support, speaking and lecture fees, and travel reimbursement. Christopher Hayward reports a relationship with Venstramedical that includes consulting or advisory. The other authors declare no competing interest.

There were no sources of funding for this research.

## References

[1] Rao P., Khalpey Z., Smith R., Burkhoff D., Kociol R.D. (2018). Venoarterial extracorporeal membrane oxygenation for cardiogenic shock and cardiac arrest. Circ Heart Fail.

[2] Makdisi G., Wang I.W. (2015). Extra corporeal membrane oxygenation (ECMO) review of a lifesaving technology. J Thorac Dis.

[3] King C.S., Roy A., Ryan L., Singh R. (2017). Cardiac support: emphasis on venoarterial ECMO. Crit Care Clin.

[4] Donker D.W., Sallisalmi M., Broomé M. (2021). Right–left ventricular interaction in left-sided heart failure with and without venoarterial extracorporeal membrane oxygenation support—a simulation study. ASAIO J.

[5] Burkhoff D., Sayer G., Doshi D., Uriel N. (2015). Hemodynamics of mechanical circulatory support. J Am Coll Cardiol.

[6] Dickstein M.L. (2018). The starling relationship and veno-arterial ECMO: ventricular distension explained. ASAIO J.

[7] Grandin E.W., Nunez J.I., Willar B. (2022). Mechanical left ventricular unloading in patients undergoing venoarterial extracorporeal membrane oxygenation. J Am Coll Cardiol.

[8] Tepper S., Masood M.F., Baltazar Garcia M. (2017). Left ventricular unloading by impella device versus surgical vent during extracorporeal life support. Ann Thorac Surg.

[9] Azimi M., Liao S., Vatani A., Burrell A., Gregory S.D. (2022). Improved flow dynamics of extracorporeal membrane oxygenation via design modification of dual-lumen cannulas. ASAIO J.

[10] Farag J., Stephens A.F., Juene Chong W., Gregory S.D., Marasco S.F. (2022). Intra-aortic balloon pump use with extra corporeal membrane oxygenation—a mock circulation loop study. ASAIO J.

[11] Stephens A.F., Wanigasekara D., Pellegrino V.A. (2021). Comparison of circulatory unloading techniques for venoarterial extracorporeal membrane oxygenation. ASAIO J.

[12] Stephens A.F., Wickramarachchi A., Burrell A.J.C., Bellomo R., Raman J., Gregory S.D. (2022). Hemodynamics of small arterial return cannulae for venoarterial extracorporeal membrane oxygenation. Artif Organs.

[13] Shehab S., Allida S.M., Davidson P.M. (2017). Right ventricular failure post LVAD implantation corrected with biventricular support: an in vitro model. ASAIO J.

[14] Cappon F., Wu T., Papaioannou T., Du X., Hsu P.L., Khir A.W. (2021). Mock circulatory loops used for testing cardiac assist devices: a review of computational and experimental models. Int J Artif Organs.

[15] Ostadal P., Mlcek M., Kruger A. (2015). Increasing venoarterial extracorporeal membrane oxygenation flow negatively affects left ventricular performance in a porcine model of cardiogenic shock. J Transl Med.

[16] Hála P., Mlček M., Ošťádal P. (2020). Increasing venoarterial extracorporeal membrane oxygenation flow puts higher demands on left ventricular work in a porcine model of chronic heart failure. J Transl Med.

[17] Brinker J.A., Weiss J.L., Lappé D.L. (1980). Leftward septal displacement during right ventricular loading in man. Circulation.

[18] Damiano R.J., La Follette P., Cox J.L., Lowe J.E., Santamore W.P. (1991). Significant left ventricular contribution to right ventricular systolic function. Am J Physiol.

[19] Summer W.R., Permutt S., Sagawa K., Shoukas A.A., Bromberger-Barnea B. (1979). Effects of spontaneous respiration on canine left ventricular function. Circ Res.

[20] Weyman A.E., Wann S., Feigenbaum H., Dillon J.C. (1976). Mechanism of abnormal septal motion in patients with right ventricular volume overload: a cross-sectional echocardiographic study. Circulation.

