# Supplementary material for: Afterload pressure and left ventricular contractility synergistically affect left atrial pressure during veno-arterial ECMO
Source: JHLT Open. 2023 Dec 14;3:100044. doi: 10.1016/j.jhlto.2023.100044 (PMC11935323; doi:10.1016/j.jhlto.2023.100044)
Supplement: Supplementary file 1 — Supplementary material [file mmc1.docx]

# Supplementary Appendix

Table S1. Experimental Protocol

| Experiment | State | ECMO Flow Direction | Starting Conditions | ECMO Flow Rate (L/min) | Procedure | Conclusions |
| --- | --- | --- | --- | --- | --- | --- |
| 1 | - Normal - LVF - RVF - BVF | - Retrograde | - mAoP: 50mmHg, 70mmHg, 90mmHg | - 0 - 2 - 3 - 4 - 5 | - Increased ECMO flow rate by adjusting only pump speed - mAoP not kept constant | - LAP increases linearly with increased mAoP; slope of LAP-AoP relationship is steeper for LV Impaired compared to LV Normal - LAP decreases linearly with increased RAP; slope of LAP-RAP relationship is steeper for LV Impaired compared to LV Normal - Numerical but not statistically significant increase in LAP with increased ECMO flow when SVR is held constant and mAoP is uncontrolled - AoP and LV gain, but not RAP or ECMO flow rate, were independent predictors of LAP |
| 2 | - Normal - LVF - RVF - BVF | - Retrograde | - mAoP: 50mmHg, 70mmHg, 90mmHg | - 0 - 2 - 3 - 4 - 5 | - Increased ECMO flow rate by adjusting pump speed and SVR - mAoP kept constant at 50, 70 and 90mmHg respectively | - No corresponding increase in LAP with increased ECMO flow when SVR is varied to allow constant mAoP |
| 3 | - LVF | - Retrograde | - mAoP: 70mmHg - LV gain: 2.4, 2.2, 2.0, 1.8, 1.6 | - 0 - 2 - 3 - 4 - 5 | - Increased ECMO flow rate by adjusting only pump speed - mAoP not kept constant | - Slope of LAP-AoP relationship increases with decreasing LV contractility |
| 4 | - LVF | - Retrograde - Antegrade | - mAoP: 70mmHg | - 0 - 2 - 3 - 4 - 5 | - Increased ECMO flow rate by adjusting only pump speed - mAoP not kept constant | - No significant difference in LAP, mAoP or total CO between retrograde and antegrade ECMO return flow - Statistically but not clinically significant increase in ECMO flow with antegrade return compared to retrograde return |

AoP = aortic pressure; BVF = biventricular failure; CO = cardiac output; ECMO = extra-corporeal membrane oxygenation; LAP = left atrial pressure; LV = left ventricle; LVF = left ventricular failure; mAoP = mean aortic pressure; RAP = right atrial pressure; RVF = right ventricular failure; SVR = systemic vascular resistance; VA = veno-arteria
